# A Comparative Study on the Integration of Eye-Tracking in Recommender Systems

**DOI:** 10.3390/s25092692

**Published:** 2025-04-24

**Authors:** Osamah M. Al-Omair

**Affiliations:** Department of Management Information Systems, King Faisal University, Hofuf 31982, Saudi Arabia; oalomair@kfu.edu.sa

**Keywords:** recommender systems, human–computer interaction, adaptive systems, eye tracking, behavioral indicators

## Abstract

This study investigated the integration of eye tracking technologies in recommender systems, focusing on their potential to enhance personalization, accuracy, and user engagement. Eye tracking metrics, including fixation duration and gaze patterns, provide a non-intrusive means of capturing real-time user preferences, which can lead to more effective recommendations. Through a comprehensive comparison of current studies, this paper synthesizes findings on the impact of eye tracking across application domains such as e-commerce and media. The results indicate notable improvements in recommendation accuracy with the use of gaze-based feedback. However, limitations persist, including reliance on controlled environments, limited sample diversity, and the high cost of specialized eye tracking equipment. To address these challenges, this paper proposes a structured framework that systematically integrates eye tracking data into real-time recommendation generation. The framework consists of an Eye Tracking Module, a Preferences Module, and a Recommender Module, creating an adaptive recommendation process that continuously refines user preferences based on implicit gaze-based interactions. This novel approach enhances the adaptability of recommender systems by minimizing reliance on static user profiles. Future research directions include the integration of additional behavioral indicators and the development of accessible eye tracking tools to broaden real-world impact. Eye tracking shows substantial promise in advancing recommender systems but requires further refinement to achieve practical, scalable applications across diverse contexts.

## 1. Introduction

Not all users behave similarly when browsing e-commerce or media websites. Despite advancements in personalization, many recommender systems still rely heavily on historical behaviors, such as purchase and browsing history, or demographic similarities to generate recommendations. This narrow data reliance creates a gap between what users truly want and what systems can infer.

Traditional recommender systems utilize methods such as collaborative filtering, content-based filtering, and hybrid models. While these techniques have improved content delivery, they often overlook rich, real-time behavioral cues that could reveal user preferences more effectively.

Recent work has shown that integrating behavioral indicators, such as eye tracking and emotion recognition, can enhance personalization by capturing implicit user signals that are not reflected in historical data [1]. Among these, eye-tracking offers a particularly promising, non-intrusive method for detecting user attention and interest through gaze patterns.

When combined with recommender systems, eye tracking can bridge the gap between observable behavior and latent user preferences, improving the relevance and timeliness of recommendations. However, despite growing interest in gaze-based personalization, existing research lacks a structured, unified approach for integrating eye tracking into real-time recommendation processes.

This paper reviews current efforts to integrate eye-tracking in recommender systems and proposes a structured framework to enhance their adaptability and personalization. Through a systematic review of the literature, we synthesize findings from various domains and recommend a modular approach that leverages gaze data to refine user modeling and improve recommendation accuracy.

## 2. Background and Related Work

### 2.1. Recommender Systems Overview

Recommender systems have gained significant popularity over the years, becoming essential tools for major corporations seeking to target users with personalized products or content. These systems typically rely on browsing and purchase history, demographic information, or other explicit data points to generate recommendations.

The most widely used approaches include collaborative filtering, content-based filtering, and hybrid methods. Collaborative filtering relies on the preferences of similar users, often within the same demographic groups [2]. Feedback typically includes user interactions such as purchases, items added to wish lists, or expressed preferences (e.g., likes). In contrast, content-based filtering uses a user’s past interactions with specific types of content to build a preference profile. One study analyzed web page content to construct a personalized model [3]. Hybrid methods combine both approaches to enhance performance and reduce limitations inherent in either one.

Beyond user-facing applications, recommendation systems are also pivotal in software engineering tasks. For instance, DDASR (Deep Diverse API Sequence Recommendation) addresses the challenge of recommending both popular and less frequent APIs by leveraging large language models and clustering techniques to enhance diversity without compromising accuracy [4]. Similarly, homogeneous graph neural networks have been employed for third-party library recommendations, capturing intricate relationships between software components to improve recommendation relevance [5]. These methods, while differing in data sources and objectives, reflect the broader versatility and evolving architecture of recommendation system technologies.

### 2.2. Eye Tracking Technology and Metrics

Eye tracking captures implicit behavioral signals by monitoring visual attention through gaze patterns and fixation metrics, offering a non-intrusive method for inferring user interest and cognitive focus. Its applications span various domains, including user interface design, advertising, cognitive psychology, and assistive technologies. In UI design, eye tracking is used to position visual elements for better usability and engagement [6]. Marketing researchers use it to assess ad visibility and viewer focus [7], while cognitive psychologists employ it to study attention, perception, and memory [8].

The technology has also advanced to support gaze-based interaction systems and assistive tools for individuals with disabilities [9]. These systems enable users to control digital interfaces, virtual environments [10], games [11], and even cars [12] using only eye movements. Assistive technologies leverage eye tracking for communication and interface control [13], offering accessibility solutions for users with motor impairments.

Beyond technical applications, eye tracking also provides a window into cognitive processes such as attention and decision making. When combined with other behavioral indicators like mouse tracking, it offers a more comprehensive understanding of user behavior. These qualities make it a valuable tool for integration with recommender systems.

### 2.3. Prior Studies Integrating Eye Tracking in Recommender Systems

A growing number of studies have examined the use of eye tracking in recommender systems, aiming to improve recommendation accuracy and user satisfaction by capturing implicit feedback through gaze data. These studies typically fall into two categories: those that use eye tracking data as a feedback signal to refine recommendation algorithms, and those that analyze user attention to assess the effectiveness of recommendation interfaces. In both cases, eye tracking serves as a behavioral indicator for inferring user interest, often measured through gaze duration, fixation count, or gaze transition patterns.

Across various domains, including e-commerce, media, and music, eye tracking has been shown to enhance personalization in meaningful ways. For example, some systems have used fixation metrics to build user interest models that improve content ranking or product placement [8], while others have integrated gaze prediction models into collaborative filtering techniques to increase accuracy [14,15]. Adaptive user interfaces have also been developed to respond in real time to gaze behavior, enabling more responsive recommendation environments [10,12]. Despite these promising developments, a number of recurring limitations are observed across the literature. Many studies rely on controlled lab-based experiments with limited ecological validity, reducing their applicability to real-world user contexts. Others depend on high-cost, specialized equipment such as Tobii or Gazepoint trackers, which poses challenges for scalability and broader deployment. Additionally, sample sizes tend to be small and demographically narrow, limiting the generalizability of the findings.

Another key limitation lies in the lack of a unified framework for integrating eye tracking into recommendation pipelines. While several approaches have demonstrated that gaze data can improve prediction quality or interface usability, few have proposed structured, real-time solutions that continuously adapt to user behavior. This gap underscores the need for a more systematic integration strategy that moves beyond isolated experiments. The present study addresses this gap by synthesizing the current literature and proposing a modular framework for incorporating eye tracking data into adaptive recommender systems.

A summary of the key studies reviewed, including their goals, domains, eye tracking metrics, recommendation strategies, key findings, and limitations are presented in Section 4. To strengthen the connection between the literature and our proposed Eye-Tracking-Based Adaptive Recommendation System framework (Section 5), we later reference representative studies in relation to each of the three framework modules. The following section outlines the systematic methodology used to identify, screen, and select these studies, ensuring the rigor and transparency in the review process.

## 3. Review Methodology

This systematic review focuses on the integration of eye-tracking in recommender systems. We followed PRISMA guidelines to identify, screen, and analyze relevant literature. Our search included the use of academic databases such as IEEE Xplore, ACM Digital Library, and Google Scholar. The keywords that were used in our search are “eye-tracking”, “recommender systems”, “predicting user preference”, and their variations. We focused on studies applying eye-tracking to recommender systems from the past two decades. Most of the reviewed literature aimed to utilize eye tracking technologies to improve the recommendation process, although a few focused on evaluating existing recommendation methodologies.

Furthermore, manual searches through the reference lists of relevant articles were conducted to identify additional studies that may have been overlooked in the initial search. Overall, 218 articles were identified during these searches, representing the first stage of the Preferred Reporting Items for Systematic Reviews and Meta-Analyses (PRISMA) framework [16]. During the second stage, i.e., screening, 123 articles were excluded due to duplication and irrelevance, leaving 95 articles for further assessment. Subsequently, in the eligibility stage of PRISMA, 42 articles were excluded after a full-text review. Many of these exclusions were due to unrelated topics, such as studies not addressing recommender systems or eye tracking. Additional exclusions included articles of poor publication quality, such as non-peer-reviewed studies, and outdated research superseded by more recent findings. At the end of this stage, 53 articles were retained for further review.

**Figure 1:**
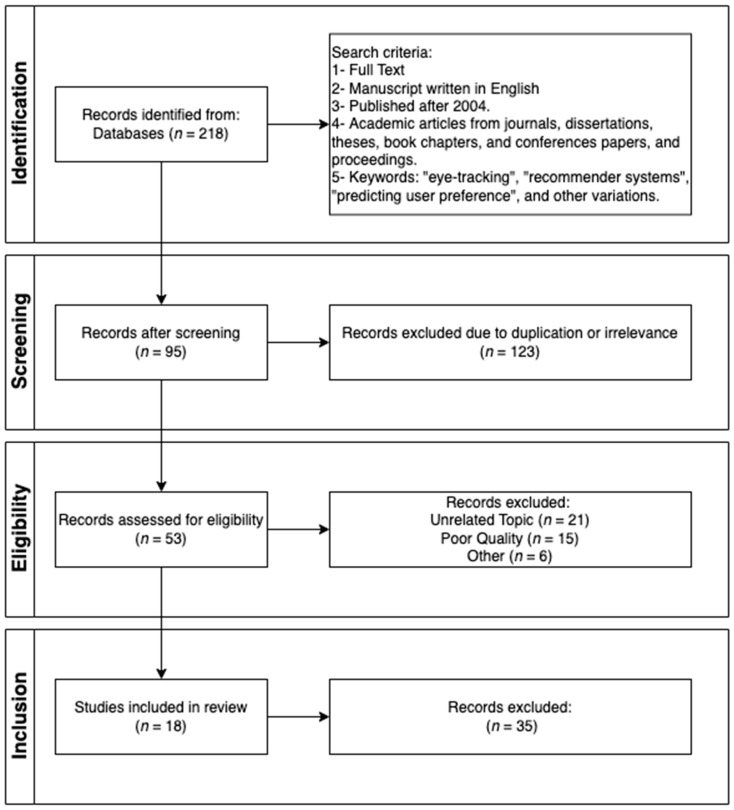
The systematic process for selecting the relevant literature reviewed in this study based on the PRISMA framework, as in [16].

In the final stage, inclusion, we conducted a thorough review of the remaining articles, selecting only high-quality publications that met our predefined criteria. This ensured that the included studies aligned with the scope and objectives of the review. To maintain the integrity and reliability of the process, only rigorous and relevant studies were included. Ultimately, 18 articles were retained for inclusion in this review. The steps of the PRISMA process are illustrated in Figure 1. This comprehensive approach allowed us to gather a diverse range of studies covering various recommendation domains, such as e-commerce, media and education where eye tracking technologies have been applied to enhance recommender system performance.

After an immense review of the collected literature, the studies were categorized based on various attributes that are relevant to our study and research goals. These attributes included domain, research goals, eye tracking technologies used, eye tracking metrics, recommendation methods, experimental design, key findings, and limitations. The majority of the reviewed literature answered all the attribute criteria, with only a few deviations. For example, domain refers to the application area of the study, such as recommender systems in e-commerce, music, or movies. Each study presented clear research goals with some diversity among them. Most used proprietary eye tracking technologies, such as high-tech eye trackers, and some only used generic webcams. Eye tracking metrics varied among the reviewed literature making it one of the most valuable attributes. Comparing the outcomes of the studies based on what eye metrics were used will aid in realizing which metrics are best for predicting user preferences.

The technological approaches of the reviewed literature were also analyzed. Specifically, the integration of eye tracking technologies with recommender systems and the various methods of recommender systems such as content-based filtering, collaborative filtering, and hybrid methods. Subsequently the findings from analyzing the reviewed studies were synthesized to identify emerging trends, overall challenges, and opportunities within the field. For future work, we plan to explore additional human indicators such as facial expressions and personality traits to be combined with eye tracking to enhance overall user experience and recommendation accuracy.

## 4. Review Findings

This section gives an in-depth analysis comparing the reviewed studies. As described in the previous section, our comparative study is based on a number of comparison attributes. An overview of study domains, goals, and methods is presented in Table 1, while key findings and limitations across the studies are summarized in Table 2. The following subsections are organized based on the comparison attributes, which evaluate and compare the studies, providing a detailed analysis across all dimensions.

### 4.1. Application Domains

The general goal of the majority of the reviewed literature is the improvement of recommender systems using eye-tracking technology. However, each has a more detailed and specific goal depending on the domain and the intended application of eye tracking data. For instance [14,29], both focused on enhancing recommendation systems by leveraging eye tracking data, but in different contexts. The study in [14] revolved around online content (documents, images, and videos), using fixation-based metrics to predict user interest and enhance recommendations. In contrast, the research in [29] targeted product recommendations in e-commerce, particularly emphasizing user satisfaction and reducing interaction effort through eye gaze data.

Similarly, the studies in [19,25] aimed to enhance e-commerce and visual analytics interfaces, respectively, using eye tracking data to model user preferences. While the research in [19] focused on adaptive user interfaces for digital camera recommendations, the study in [25] focused on visualizing time-series patterns in data analytics, making their application of eye tracking highly domain-specific. Despite these differences, both studies share the goal of making their systems more adaptive and personalized by utilizing eye tracking data.

The variation in domain creates a unique context for each study. For instance, e-commerce studies by [22,33] focus more on product presentation and how layout influences user behavior, while [26] examines the impact of image-based interfaces on gaze transitions. This diversity allows for a broader understanding of how eye tracking data can be applied across different environments. The study in [23] incorporates gaze prediction to better understand user preferences to enhance movie recommendations. Similarly, ref. [32] study personal characteristics in music recommendation systems using eye tracking data, revealing a shift in focus toward user behavioral profiling and personalization.

The overall consensus in the shared goals of the studies in this review is to utilize eye tracking to predict user preference to generate more desirable recommendations, thus improving recommendation accuracy and overall user satisfaction. On the contrary, some of the studies use eye tracking to measure other proposed methods for improving the recommendation process such as timing, layout, and positioning. The studies also differ in the technologies and methods used for eye tracking, which will be discussed in the following subsection.

### 4.2. Eye Tracking Metrics

The reviewed studies employed a range of eye tracking techniques and metrics, reflecting both methodological diversity and recurring patterns in measurement, such as fixation duration and gaze transitions. Tobii devices (1750, T60, and X2-60) were frequently used among the studies, suggesting that Tobii eye trackers have become a standard for capturing gaze data in these types of experiments. For example, Tobii 1750 was used in studies by [7,12,13] to capture fixation frequency and duration, while Tobii T60 was used by [23,24] for more advanced gaze prediction and user behavior analysis. Other studies used standard webcams, such as [27], to measure the gazing level. Although using a standard webcam is more accessible to the general public, as discussed in [35], it causes limitations in accuracy. Studies commonly used fixation duration, frequency, and gaze transitions to infer attention and predict preferences. For example, ref. [23] employed fixation probability and duration to improve gaze prediction models, whereas [9,17] analyzed fixation frequency to compare user behavior across different interfaces. One unique metric in [19] was pupil diameter, which was used to measure user arousal levels. This highlights a shift from purely gaze-based metrics to more physiological measures of attention.

A key difference lies in the depth of eye tracking analysis. For example, ref. [32] used a more sophisticated array of metrics to explore behavioral traits in a music recommendation context that includes saccade velocity and direction. This contrasts with simpler approaches like [25], which relied on basic fixation metrics to predict patterns in time-series data visualizations. More advanced metrics can provide deeper insights into user behaviors, but may also introduce complexity in interpretation and additional computational cost.

### 4.3. Recommendation Methods

The studies utilize a range of recommendation algorithms, from content-based filtering to collaborative filtering and even interactive genetic algorithms. However, these techniques are combined with eye-tracking data for different purposes, but mainly to enhance the relevance and accuracy of recommendations that are tailored to user preferences. For instance, ref. [29] used both content-based and collaborative filtering to recommend products based on eye gaze data as implicit feedback, leading to more accurate and user-satisfying product recommendations.

The authors in [14] implemented a content-based filtering method driven by attention time, which was derived from fixation points and durations. This method relied on implicit feedback gathered through video oculography, which helped determine content relevance by assessing how long users visually engaged with certain items. The results indicated that this eye-tracking-driven approach offered a significant improvement over traditional recommendation techniques, establishing that attention time is a reliable predictor of interest. Similarly, ref. [23] applied collaborative filtering enhanced with gaze prediction models, including logistic regression and Hidden Markov Models (HMMs). This hybrid approach, combining implicit feedback with predictive models, demonstrated that eye tracking data can be effectively generalized across users to refine collaborative filtering systems.

Content-based filtering using Constrained Dynamic Time Warping (CDTW) was introduced by [25] to develop a visual analytics recommendation model. By pairing eye gaze metrics with time series data, this system suggested patterns for users to explore, improving the relevance of visualizations for data analysis tasks. This approach underscored the utility of eye tracking in areas beyond conventional product recommendations, as the combination of time series pattern recognition and gaze data led to a more adaptive and personalized user experience.

In a different approach, ref. [27] integrated eye tracking and emotion recognition for more customized product recommendations without depending on historical data, making the system more personalized and adaptive. A content-based filtering model was developed that combined eye gaze data with emotion detection through facial expressions. Using webcam-based gaze tracking and emotion analysis, this system aimed to deliver product recommendations without historical user data. The integration of eye tracking and emotion recognition yielded a 76.6% accuracy rate in recommendations, suggesting that implicit feedback on both visual attention and emotional responses could help tailor recommendations effectively.

A more innovative method was proposed by [19], which used an Interactive Genetic Algorithm (IGA) to optimize recommendations based on eye tracking data in an e-commerce context. This method leveraged eye tracking metrics, including gaze percentage and pupil diameter, to build adaptive recommendations. The Interactive Genetic Algorithm (IGA) is a type of optimization that uses user feedback to evolve solutions. In this study, IGA worked with eye tracking data, using indicators like where and how long users looked at product features to predict preferences. Based on this implicit feedback, the IGA adjusted its recommendations to show products that matched users’ interests more closely without needing direct user input. This approach proved effective in improving recommendation accuracy, achieving high user satisfaction and outperforming traditional recommendation methods in their experiment.

Some studies, such as [7,26], explore the impacts of different user interfaces on gaze behavior without directly applying eye tracking data in their recommendation algorithms. Similarly, refs. [15,20] use eye tracking to study user behavior in addition to interface effectiveness. These studies highlight that eye tracking may not necessarily be directly integrated into recommendation algorithms, but it can significantly influence a system’s design. This limits the direct application of eye tracking to the recommendation process but provides valuable insights into how users interact with different interfaces.

The authors in [15] applied a preference-based organization technique to examine how different layouts influenced user attention during e-commerce interactions. Although eye tracking data were not directly incorporated into generating recommendations, it was used to analyze user engagement across different interfaces, leading to insights on how layout design can affect product selection. Similarly [13], explored diversity-based recommendations in the perfume shopping domain, using collaborative filtering and editorial-picked critiques to broaden user exposure to different products. Eye tracking data measured engagement but were not directly involved in generating recommendations. The diversity-based approach enhanced user satisfaction, as it provided a variety of options that catered to different preferences, although it sometimes came at the cost of recommendation accuracy.

The reviewed studies illustrate how diverse recommendation methods, from content-based filtering and collaborative filtering to genetic algorithms and time series analysis, can benefit from eye tracking data as a source of implicit user feedback. Despite methodological differences, the consistent use of eye tracking highlights its potential to refine recommendations by capturing nuanced aspects of user interest that traditional methods alone may miss.

### 4.4. Experimental Designs and Key Findings

The experimental designs and key findings across these studies reveal a range of approaches to incorporating eye tracking data into recommendation systems, demonstrating its potential to improve recommendation accuracy and user satisfaction. Most experiments were conducted in controlled lab settings, providing a structured environment to monitor eye movements and evaluate the impact of different recommendation methods. This design enabled studies to isolate the effects of eye-tracking metrics, such as fixation duration, gaze paths, and pupil dilation, on recommendation accuracy. Studies such as [14,23] used lab-based setups to assess how fixation metrics corresponded with user interest as participants viewed digital content or movies. However, this controlled approach also introduced limitations, as the findings may not fully generalize to real-world, varied conditions due to the lack of environmental and behavioral diversity.

For [14], a lab-based study was conducted to test the effectiveness of content-based filtering using eye tracking data to determine attention time, which was used to refine recommendations for online documents, images, and videos. This approach showed that attention time, measured through fixation points, is a reliable predictor of user interest that resulted in improved recommendation quality compared to traditional methods. However, the study’s use of generic eye tracking devices raised concerns about accuracy, and the sample lacked diversity, limiting the applicability of the findings across broader contexts.

A significant number of the studies utilized video oculography, often relying on specialized devices like the Tobii eye tracking monitors, to capture nuanced metrics such as gaze probability and fixation frequency. In the e-commerce domain, studies such as [19] focused on adaptive user interfaces for e-commerce, specifically in recommending digital cameras. Here, eye tracking metrics like fixation duration, gaze percentage, and pupil diameter informed an Interactive Genetic Algorithm (IGA) to create user-centered recommendations. This experimental design aimed to optimize recommendations dynamically through an adaptive interface. The results were promising, with an accuracy of 87.5% in recommendations, but a small sample size and limited participant diversity highlighted potential generalizability issues. In addition, utilizing specialized eye trackers facilitated more accurate data collection but also limited the scalability of these experiments due to the cost and expertise required for such equipment.

In [20], the experimental focus shifted to the role of diversity in recommendations, especially within the specific domain of perfume shopping. Their lab-based study indicated that diverse recommendations increased user satisfaction and reliance on the system, even though this diversity slightly decreased recommendation accuracy. The findings suggest that adding variety to recommendations may support user decision making, although the results may be less applicable to other domains with different consumer behaviors.

In the context of movie recommendations, ref. [23] used eye tracking data to develop a collaborative filtering model enhanced by gaze prediction. By using logistic regression and Hidden Markov Models (HMMs), they accurately predicted fixation probability and duration, which supported higher recommendation accuracy than a browsing-data-only approach. The study’s controlled environment and the inclusion of a small, non-diverse sample, however, may restrict the relevance of the findings to broader, real-world applications.

The authors of [24] explored how the timing and source of recommendations (expert vs. consumer reviews) impacted user interest in e-commerce products. Their findings showed that consumer-generated recommendations elicited more interest than expert opinions, as measured through pupil dilation. However, fixation duration was not significantly affected by the timing of the recommendations, suggesting that attention levels were relatively stable across recommendation stages.

The key findings across these studies consistently support the potential of eye-tracking data to enhance recommendation system accuracy. In many cases, studies demonstrated that fixation-related metrics, such as attention time and frequency of fixations, aligned closely with user interest, validating the use of eye tracking as an implicit feedback tool. For example, refs. [14,34] found that increased attention time on specific items improved the accuracy of content-based and collaborative filtering recommendations. Additionally, studies in movie and e-commerce contexts showed that fixation duration and gaze distribution patterns could predict interest effectively, providing a non-intrusive alternative to traditional feedback mechanisms.

Despite promising results, the findings also highlight limitations, primarily due to small sample sizes and the specificity of the tested domains. For example, the perfume shopping behaviors studied by [20] may not generalize to other product categories, as users’ browsing patterns vary significantly by domain. Similarly, the demographic constraints of the studies, often limited to young or homogeneous groups, limit the applicability of the results to broader populations. These limitations underscore the need for future research to conduct more ecologically valid studies with larger, more diverse samples and real-world environments to strengthen the generalizability of the findings. The studies reviewed demonstrate the potential of eye tracking in recommender systems but lack a structured approach that fully leverages gaze-based features for real-time preference adaptation. To address this limitation, we propose a novel framework that integrates eye tracking data into an adaptive recommendation process discussed in the following section.

## 5. Proposed Framework: Eye-Tracking-Based Adaptive Recommendation System

**Figure 2:**
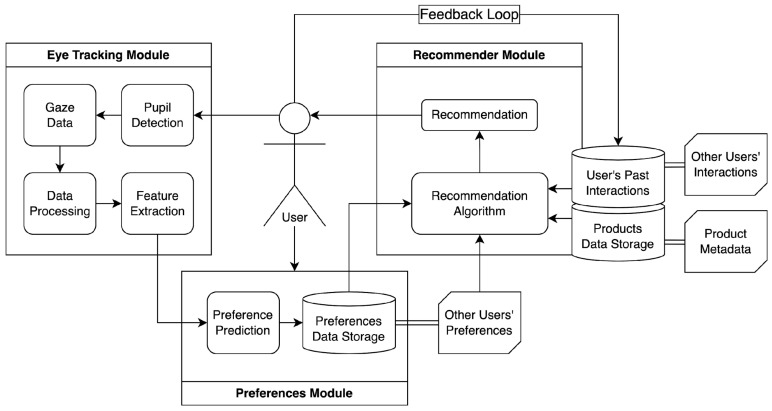
The Eye-Tracking-Based Adaptive Recommendation System Framework.

While previous studies have explored the integration of eye tracking in recommender systems, there remains a gap in designing a unified framework that systematically incorporates gaze-based preferences into real-time recommendation generation. To address this, we propose a structured framework that enhances the adaptability and responsiveness of recommender systems by leveraging eye-tracking data shown in Figure 2. The proposed framework integrates an Eye Tracking Module, a Preferences Module, and a Recommender Module to create an adaptive recommendation system that dynamically adjusts based on real-time gaze data and user interactions.

The findings discussed in the previous section map directly onto the components of the proposed Eye-Tracking-Based Adaptive Recommendation System framework. Specifically, the reviewed studies related to gaze capture techniques and visual metrics support the Eye-Tracking Module, those that infer user preferences from behavioral signals relate to the Preference Inference Module, and works focusing on content delivery and algorithmic adaptation inform the Recommendation Module. This mapping reinforces the practical relevance and theoretical grounding of each framework component. Each module in the proposed framework is grounded in evidence from the reviewed studies, as discussed in the subsections that follow.

### 5.1. Eye Tracking Module

This module captures gaze data and pupil detection signals from the user. The collected data undergoes processing and feature extraction to identify key indicators of user engagement, interest, or fatigue. These features form the basis for understanding user preferences beyond explicit interactions.

The design of this module is supported by several studies that rely on gaze-based features such as fixation duration, saccade patterns, and pupil dilation to capture visual attention and cognitive load [8,15,24]. These metrics have been shown to effectively reflect user engagement and decision-making processes in recommendation environments, providing the foundation for real-time attention modeling.

### 5.2. Preferences Module

The extracted features from the Eye Tracking Module are fed into the Preference Prediction component, which generates dynamic user profiles by analyzing gaze-derived signals in conjunction with historical preferences and inferred behavioral patterns. This updated preference data is stored alongside historical preferences and other users’ preference patterns, enriching the system’s ability to predict user intent with greater accuracy.

Prior work has demonstrated that gaze behavior can be translated into implicit feedback for modeling user preferences. For instance, ref. [15] used gaze duration to predict item interest, while [32] incorporated gaze heatmaps into preference models. These studies support the use of eye tracking data as a behavioral proxy for interest, making it a valuable input for dynamic user profiling.

### 5.3. Recommender Module

The recommendation algorithm leverages multiple data sources, including user past interactions, product metadata, and other users’ interactions, to generate real-time recommendations. The system continuously updates recommendations via a feedback loop, incorporating both explicit (clicks, selections, ratings) and implicit (eye movement, fixation duration, fixation count) behavioral signals.

This component is informed by studies that integrate eye-tracking signals into content re-ranking algorithms and adaptive interfaces. For example, refs. [10,12] explored systems that modified content layout based on gaze interaction, and [32] used gaze-enhanced collaborative filtering to improve recommendation accuracy. These examples validate the feasibility of real-time, gaze-driven personalization strategies.

By integrating real-time physiological signals, the framework enhances recommendation relevance by dynamically adapting to changing user interests and reducing reliance on static user profiles through continuous gaze-based feedback. This approach allows for a more personalized and context-aware recommendation process, distinguishing it from conventional recommender systems. To evaluate the practicality and relevance of the proposed framework, the following discussion reflects on its alignment with existing findings and highlights areas for future exploration.

## 6. Discussion

This review highlights the substantial role of eye tracking in enhancing recommender systems through improved recommendation accuracy, personalization, and user engagement. Across the reviewed studies, eye tracking metrics consistently provide valuable insights into user preferences and interests. These metrics enable systems to capture implicit feedback in a non-intrusive way, allowing for dynamic recommendations that more accurately reflect user engagement. However, existing systems lack a structured approach for incorporating gaze-based feedback into real-time recommendation updates. The proposed framework addresses this gap by integrating an Eye Tracking Module to continuously capture and process gaze data, a Preferences Module to refine user interest modeling, and a Recommender Module that dynamically adapts recommendations based on implicit gaze interactions. For instance, in [14], attention time based on fixation points improved recommendation relevance, while [19] used gaze percentage and fixation duration to tailor adaptive e-commerce interfaces, achieving notable accuracy improvements. Studies such as [23] have also found that eye-tracking significantly enhances gaze prediction in collaborative filtering models, thereby improving the alignment between user interest and recommendations.

While the reviewed studies generally support the value of eye-tracking for enhancing recommender systems, there are notable differences in the degree of reported improvements in recommendation accuracy and user satisfaction. These inconsistencies can be attributed to several contextual and methodological factors. For instance, studies conducted in highly controlled lab settings often report higher accuracy gains [15,32], while those using naturalistic environments tend to face more noise and variability [8]. Additionally, variation in sample characteristics, such as participant expertise, age, or familiarity with the platform, can influence gaze behavior and preference patterns. Differences in the precision and calibration of eye-tracking devices, as well as the way gaze data is integrated into recommendation algorithms, also contribute to discrepancies in results. Recognizing these factors is essential for interpreting outcomes and assessing the generalizability of findings across domains and user populations.

In addition to methodological differences, limited sample diversity also affects the generalizability of existing findings. Many of the reviewed studies rely on relatively young, tech-savvy participants, often university students, which may not represent the broader user population. However, user behavior can vary significantly with age, cultural background, and technical proficiency. For example, older users may exhibit different gaze patterns or interact with recommendation interfaces more cautiously [8], while users from different cultural contexts may interpret visual cues differently or rely more on certain types of information [15]. Prior research has shown that age and experience substantially influence how users navigate and engage with digital systems [36]. As such, conclusions drawn from homogeneous samples should be interpreted with caution, and future research should prioritize more inclusive sampling to ensure broader applicability.

Despite these promising findings, several limitations were observed across the reviewed studies. A key limitation is the reliance on controlled, lab-based experimental settings, which reduces the applicability of findings to real-world scenarios. Similarly, while the proposed framework enhances recommendation accuracy by integrating gaze-based feedback, its implementation may introduce computational challenges due to the continuous processing of real-time eye-tracking data. The need for efficient feature extraction and preference prediction algorithms must be addressed to ensure the system remains responsive without excessive processing overhead. While controlled environments provide precise measurements of eye movements, they may not capture the full range of user behaviors in real-world scenarios, where environmental variables and spontaneous interactions often influence user attention. This was evident in studies like [26], which used lab-based setups to assess gaze behaviors in movie recommendations, and [30], whose results indicated that real-world applicability might be limited by small, homogenous samples. This demographic bias limits the findings’ applicability across broader populations, particularly when adapting recommendations for varied age groups or cultural backgrounds.

Another notable constraint lies in the dependency on specialized eye tracking equipment, such as Tobii and Gazepoint devices, which can be cost-prohibitive for broader deployment. For example, ref. [27] explored the potential of webcam-based gaze tracking for product recommendations but found that accuracy was compromised compared to higher-end systems. This trade-off between data accuracy and system scalability remains a significant challenge, particularly for commercial applications where affordability and ease of implementation are crucial.

In terms of domain-specific implications, eye tracking contributes differently across fields such as e-commerce and media. In e-commerce, where product recommendations are essential for purchase decisions, eye tracking enhances interface adaptiveness. Moreover, ref. [19] showed that eye gaze patterns help align recommendations with user interest, enhancing interface responsiveness. In contrast, movie recommendations, as explored by [23], benefit from gaze-based personalization models, although these approaches are limited by domain specificity and demographic factors. Addressing these barriers requires more versatile models that can adapt eye tracking insights across varied recommendation contexts.

For real-world applications, the potential of eye tracking to refine recommendation systems is clear but requires overcoming several limitations. Future research should aim to explore field studies or hybrid experimental designs that better simulate naturalistic user behaviors, enhancing ecological validity. Additionally, the proposed framework could be extended by integrating other behavioral signals, such as facial expressions or mouse tracking, to enhance preference prediction. Combining eye-tracking with multi-modal behavioral data could improve the robustness of recommendations, making the system more adaptable across different domains. Moreover, research should investigate the trade-offs between real-time processing efficiency and recommendation quality, ensuring that gaze-based personalization remains computationally viable for large-scale applications. Expanding sample diversity and integrating alternative tracking technologies, as suggested by [27], could improve scalability.

In summary, while eye-tracking offers a valuable tool for enhancing recommender systems, practical application requires addressing challenges in generalizability, scalability, and cross-domain adaptability. Continued research into these areas will be essential to realizing the full potential of gaze-based recommendations in personalized systems.

## 7. Recommendations and Future Directions

To advance the integration of eye tracking within recommender systems, several key areas warrant focused attention. First, increasing the ecological validity of eye tracking studies is essential for enhancing the applicability of findings across real-world scenarios. This could involve conducting field studies or hybrid experimental designs that better capture spontaneous user interactions in more natural environments. Such approaches would reduce the dependency on controlled, lab-based settings and provide more generalized insights into user behaviors.

Expanding the diversity of sample populations is another critical step to improve the generalizability of eye tracking research. Many current studies focus on young, homogeneous groups, which limits the applicability of findings across diverse demographic groups. Future research should prioritize recruiting participants across varied age ranges, cultural backgrounds, and technological familiarity to better understand how different user groups interact with recommender systems. This diversity would support the development of systems adaptable to a broader audience, ultimately enhancing recommendation accuracy and user satisfaction.

Enhancing eye tracking technology for broader accessibility and scalability is also vital. To reduce reliance on high-cost, specialized eye tracking devices like Tobii systems, future studies should consider alternative technologies, such as advanced webcam-based tracking or lightweight wearable eye-trackers. While these options currently exhibit reduced accuracy compared to specialized equipment, further advancements in software algorithms, including gaze estimation and calibration techniques, could improve their precision. More accessible tracking technologies would allow for scalable applications in commercial and educational recommender systems, reaching a wider audience at a lower cost.

Incorporating hybrid recommendation methods that leverage both eye-tracking data and additional behavioral indicators is another promising direction. Studies have shown that combining eye gaze metrics with emotional cues, facial expressions, or mouse movements can enhance the accuracy and personalization of recommendations. For instance, pairing gaze data with real-time emotion analysis could create a multi-layered model of user preferences. Particularly, in applications such as media and e-commerce, where affective responses significantly influence decision-making. Researchers and practitioners should explore hybrid models that integrate eye-tracking with other implicit indicators to create more nuanced, adaptive systems.

Furthermore, prioritizing specific eye tracking metrics that have consistently demonstrated predictive value could optimize system performance. Metrics such as fixation duration, gaze transitions, and pupil dilation were frequently cited as reliable indicators of user interest. Standardizing these metrics across studies could facilitate more direct comparisons of results and identify the most effective indicators for specific recommendation tasks, such as content-based versus collaborative filtering models.

In summary, future research and practical implementations should emphasize ecological validity, sample diversity, technology accessibility, hybrid behavioral models, and standardized eye tracking metrics. These approaches hold the potential to overcome current limitations, driving meaningful advancements in recommender system accuracy and user engagement.

## 8. Conclusions and Future Works

This review has explored the integration of eye tracking technologies within recommender systems, highlighting the potential of gaze-based data to enhance personalization, accuracy, and user engagement. By capturing implicit feedback through metrics such as fixation duration and gaze patterns, eye tracking offers a non-intrusive means of understanding user preferences in real time. The studies reviewed demonstrate the promise of eye tracking to refine recommendation methods across different domains, providing valuable insights into user behavior that go beyond traditional data sources.

However, the current literature reveals several limitations that need to be addressed for eye-tracking to reach its full potential in practical applications. Overcoming current constraints such as limited diversity and lab-based settings is crucial for real-world applicability. Addressing these issues will require recruiting a more diverse range of participants, designing experiments that reflect real-world environments, and developing more accessible eye tracking technologies. These steps are essential for advancing the generalizability and scalability of eye tracking applications in practice.

To maximize the impact of eye tracking in recommender systems, future research should also consider hybrid approaches that incorporate additional behavioral indicators alongside gaze data. By combining eye-tracking with metrics like facial expression or mouse movement, recommendation systems can achieve a deeper understanding of user intent and provide more tailored, context-aware suggestions.

As the next step, we plan to conduct a pilot study using real eye tracking data to evaluate the effectiveness of the proposed framework. This will allow us to assess its practical applicability, refine its components based on empirical evidence, and better understand how it performs in real-world user scenarios. Incorporating experimental validation is a necessary phase to support the framework’s reliability and to move toward implementation in applied settings.

In summary, while eye tracking brings significant opportunities for creating more adaptive and responsive recommender systems, overcoming existing limitations is crucial to translating these advancements into practical, widely applicable solutions. Continued research in this area will not only improve system accuracy, but also enhance user experiences across diverse digital environments.

## Figures and Tables

**Table 1:** Summary of eye tracking approaches in recommender systems.

Reference	Domain	Goal	Eye Tracking Method(s)	Eye Tracking Metrics	Recommendation Method(s)
Simonetti et al. (2023) [7]	Banner Advertising	Investigate how the placement of banner ads on web pages affects user attention, and how attention relates to ad position, click behavior, and recognition.	Tobii X2-30 Compact eye tracker (Stockholm, Sweden)	- Fixation Duration - Fixation Frequency - Revisits	- Did not generate recommendations.
Xu et al. (2008) [14]	Online documents, images, and Videos	Develop an online content recommendation algorithm based on attention using eye tracking.	Opengazer [17] (standard webcam)	- Attention Time (fixation points and durations)	- Content-based filtering based on attention time using eye tracking
Chen et al. (2010) [15]	E-commerce (laptops)	Investigate the impact of different recommender interface designs on users’ decision-making strategies by analyzing eye movements and product selection behavior.	Tobii 1750 eye tracking monitor	- Fixation Frequency and Duration	- Preference-based organization [18] (eye-tracking not applied)
Cheng et al. (2010) [19]	E-commerce (digital cameras)	Develop an adaptive user interface for product recommendation that utilizes eye tracking data to model user preferences and enhance recommendation accuracy.	RS H6 Eye Tracker by ASL™ Ltd. (Nairobi, Kenya)	- Gaze Percentage - Fixation Duration Mean - Transitions - Pupil Diameter	- Eye tracking data to model user preferences - Interactive Genetic Algorithm (IGA) to optimize recommendations - Adaptive user interface (AUI) to present recommendations
Castagnos et al. (2010) [20]	E-commerce (perfumes)	Investigate the need for diversity in product recommendations and how it impacts user decision-making processes.	Tobii 1750 eye tracking monitor	- Fixation Frequency and Duration	- Diversity-Based Recommendations - Editorial Picked Critiques (EPC) [21] - Collaborative-Based Filtering
Chen et al. (2011) [22]	E-commerce (Laptops)	Analyze how different organizational layouts in recommender interfaces affect users’ eye gaze patterns and their decision-making processes.	Tobii 1750 eye tracking monitor	- Fixation Frequency - Fixation Duration - Gaze Path	- Preference-based organization [18] (eye-tracking not applied)
Zhao et al. (2016) [23]	Movies	Improve accuracy of recommender systems by incorporating gaze prediction to enhance understanding user preferences and behaviors.	Tobii T60 Eye Tracker	- Fixation Probability - Fixation Duration	- Collaborative Filtering
Shi et al. (2017) [24]	E-commerce	Investigate how timing and source of online product recommendations affect consumers’ interest and attention.	Tobii T60 Eye Tracker	- Fixation Duration - Pupil Dilation	- Collaborative Filtering
Silva et al. (2018) [25]	Visual Analytics (time series patterns)	Improve efficiency of visual analysis by developing a recommendation model that leverages eye gaze data and time series features to help users identify interesting patterns in data visualizations.	EyeTribe Eye Tracker	- Fixation Duration - Fixation Frequency	- Content-based filtering using Constrained Dynamic Time Warping (CDTW)
Gaspar et al. (2018) [26]	Movies	Investigate how different representations of recommended items influence user behavior.	Tobii X2-60 Eye Tracker	- Fixation Duration - Fixation Frequency - Fixation Sequence	- Content-based filtering
Jaiswal et al. (2019) [27]	E-commerce	Develop a recommendation system that incorporates user’s emotions and interests, captured through eye gaze and facial expressions, to provide personalized product recommendations without the need for historical user data.	Standard webcam	- Eye Gaze	- Content-based filtering using eye gaze and emotions
Song et al. (2019) [28]	E-commerce (smart TVs and smartphones)	Develop a recommendation system that combines social network data and eye tracking data to analyze user preferences and behaviors.	Standard webcam	- Gazing Level (concentration level)	- Eye tracking data - Social behavior data (collaborative filtering)
Fahim et al. (2020) [29]	E-commerce	Develop a recommendation system that implicitly uses eye gaze data to recommend products.	Standard webcam	- Fixation Duration - Fixation Frequency	- Content-based filtering using eye tracking - Collaborative Filtering using eye tracking
Jia et al. (2021) [30]	E-commerce (mobile phones and laptops)	Investigate how the timing of product recommendations affects consumers’ attention and processing of recommended information.	Tobii T60 eye tracker	- Fixation Duration - Fixation Frequency - Total Fixation Duration	- Did not generate recommendations.
Sari et al. (2021) [31]	E-commerce (hijab and women’s modest wear)	Develop a recommendation system using eye tracking data to predict consumer interest and purchasing behavior.	Eye Tribe eye tracker	- Fixation Duration	- Collaborative Filtering using eye tracking
Millecamp et al. (2021) [32]	Music	Investigate whether users’ personality traits can be classified using users’ gaze patterns during interaction with a music recommender system.	Tobii 4C remote eye tracker	- Fixation Rate - Fixation Duration - Saccades - Average Pupil Size	- Content-based filtering (Not based on eye tracking)
Sulikowski et al. (2021) [33]	E-commerce	Evaluate the effectiveness of recommendation interfaces on e-commerce websites by analyzing layout, position, and visual intensity using eye tracking and event tracking methods.	Gazepoint GP3 eye tracker (Vancouver, BC, Canada)	- Fixation Duration - Visual Intensity	- Content-based filtering (not based on eye tracking)
De Leon-Martinez et al. (2023) [34]	Movies	Investigate whether eye tracking can be used to improve the accuracy of a collaborative filtering model for movie recommendations.	Tobii X2-60 eye tracker	- Fixation Duration	- Collaborative Filtering

**Table 2:** Summary of the key findings and limitations.

Reference	Key Findings	Limitations
Simonetti et al. (2023) [7]	- Less attention was given to banner ads during goal-oriented tasks than during less goal-oriented tasks. - Banners in the middle position received more clicks, though attention levels across positions were similar in less goal-oriented tasks. - Banner ads were still highly recognized one day and one week later, demonstrating effective long-term memory retention even with minimal initial attention.	- General ads were given without a recommendation process. - The study did not assess ad relevance to participants, which may have influenced outcomes. - The experiment only tested a desktop version of the web page, and results might differ on mobile devices. - The fixed task order could have impacted the findings.
Xu et al. (2008) [14]	- Improved recommendations compared to traditional methods.	- The use of generic eye tracking devices may lack the accuracy and precision of specialized eye tracking systems. - Limitation of media types used in the study. - Lack of user diversity in the study’s test subjects.
Chen et al. (2010) [15]	- Users focused more on the top items in the list interface, leading to limited consideration of other options. - Organization-based interfaces, especially those with a quadrant layout, significantly increased the number of items viewed by users compared to the traditional list interface.	- Eye tracking was only used to test the different layout interfaces for recommended items. - Small sample size, limiting the generalizability of the findings. - Lack of user diversity in the study’s test subjects.
Cheng et al. (2010) [19]	- Achieved an accuracy of 87.5% in giving recommendations. - Obtained higher user feedback on their system compared to a traditional system.	- Small sample size, limiting the generalizability of the findings. - Lack of user diversity in the study’s test subjects. - Complexity of product features.
Castagnos et al. (2010) [20]	- The diversity-based recommender system achieved more desirable outcomes when compared to the traditional multi-criteria filtering (MFC) approach. - Diverse recommendations can enhance user satisfaction despite potentially decreasing average accuracy.	- Eye tracking was not utilized in generating recommendations. - The nature of the perfume domain may not accurately reflect user behavior in other e-commerce domains. - Small sample size. - Lack of user diversity in the study’s test subjects.
Chen et al. (2011) [22]	- The organization-based interfaces (vertical-based and quadrant-based) led to more dispersed fixation distributions compared to the traditional list interface. - The quadrant-based interface resulted in the highest percentage of users making product selections (100%) compared to the vertical-based (71.43%) and traditional list interfaces (50%).	- Eye tracking was not utilized in generating recommendations. - Small sample size, limiting the generalizability of the findings. - The use of a controlled experimental setting might not fully capture real-world user behavior. - The organization-based interfaces were designed specifically for the experiment, and their effectiveness in different e-commerce contexts may vary.
Zhao et al. (2016) [23]	- Including eye tracking data significantly improved the accuracy of gaze predictions compared to using browsing data alone. - Logistic regression and Hidden Markov Models (HMMs) were effective in predicting fixation probability and fixation time. - Gaze prediction can be effectively generalized across different users.	- Small sample size, limiting the generalizability of the findings. - The use of commodity eye tracking devices may introduce some inaccuracies compared to more sophisticated eye tracking systems.
Shi et al. (2017) [24]	- Consumer recommendations elicited greater interest (measured by pupil dilation) than expert recommendations. - The timing of recommendations did not significantly impact the fixation duration, suggesting that earlier recommendations did not receive more attention than later ones.	- Small sample size, limiting the generalizability of the findings. - Only two types of products (laptops and cell phones) were considered, which may not represent all online shopping experiences. - Excluding a personalized recommender system algorithm in the design of the study.
Silva et al. (2018) [25]	- The recommendation model combining eye gaze and time series features improved the accuracy of pattern recommendations. - The model could predict the final choices of users with a reasonable degree of accuracy. - Users found the adaptive visualizations and recommendations relevant and helpful for time-series analysis tasks. - The integration of eye gaze data enhanced the system’s ability to provide personalized and contextually relevant recommendations.	- Small sample size and specific demographic (mostly young students) limits the generalizability of the findings. - The focus was on time series patterns, which may limit the generalizability to other recommender system domains. - The complexity of integrating multiple types of data (eye gaze and time series features) requires sophisticated modeling and computational resources.
Gaspar et al. (2018) [26]	- Image-based interfaces induced different gaze behaviors compared to text-based interfaces, as participants made more frequent and larger gaze transitions between items when viewing images. - When movies were from preferred genres, participants made smaller gaze transitions, which suggests more focused attention.	- Small sample size, limiting the generalizability of the findings. - The controlled lab setting may not fully replicate real-world conditions where users interact with recommendation systems. - The use of a circular layout, while methodologically beneficial for eye tracking, is not a common interface in real-world applications and may influence the generalizability of the results.
Jaiswal et al. (2019) [27]	- The integration of eye gaze and emotion detection improved the relevance of recommendations. - Achieved an accuracy of 76.6% in giving recommendations.	- The accuracy of the system depends on the quality of the webcam and the lighting conditions. - The system may require calibration for different users to improve accuracy.
Song et al. (2019) [28]	-Achieved an accuracy of 98.5% for smart TVs and 96.5% for smartphones. - User preferences can differ depending on the device used by the user, highlighting the importance of considering device characteristics in recommendation systems. - Integrating eye tracking and social behavior data yields a more accurate understanding of user preferences than using a single data source.	- Small sample size, limiting the generalizability of the findings. - The use of a single web camera for eye tracking may have limitations in accuracy compared to more advanced eye tracking technologies.
Fahim et al. (2020) [29]	- Using eye gaze data as implicit feedback can effectively capture user interest, leading to more accurate and user-satisfying product recommendations.	- The system’s performance is limited by the quality of the collected eye gaze data and the ability to accurately interpret this data to infer user interest.
Jia et al. (2021) [30]	- Early recommendations received the most attention from participants. - Participants paid more attention to product descriptions than to recommendation signs or reviews. - Attention to recommendation signs remained relatively consistent across different recommendation times, while attention to product descriptions and reviews varied significantly.	- Small sample size, limiting the generalizability of the findings. - The experiment only included utilitarian and search products, excluding hedonic and experience products which may yield different results. - The study did not fully explore primacy and recency effects due to the nature of the experimental tasks. Further studies are needed to investigate these effects.
Sari et al. (2021) [31]	- The product recommendations based on eye tracking data provided relevant recommendations. - The use of real-time consumer attention data helped address issues of sparsity and cold start in traditional recommendation systems.	- The study was limited to a specific type of e-commerce site, which may not generalize to other types of products or broader e-commerce contexts. - The accuracy of predictions depends heavily on the quality and quantity of eye tracking data collected, which may vary with different users and sessions.
Millecamp et al. (2021) [32]	- The study found that it was possible to classify users’ need for cognition (NFC) using Logistic Regression with higher accuracy than a random baseline. - Openness could be classified more accurately than the baseline using the Gradient Boosting classifier, particularly in the early stages of the task. - Musical sophistication could not be classified better than the baseline using the gaze data.	- The classification accuracy for NFC and openness was not high enough for practical use in adapting the recommender system’s interface. - The study did not include areas of interest (AOI) related features, which may have affected the classification results, particularly for musical sophistication.
Sulikowski et al. (2021) [33]	- Vertical layout was more effective in driving purchases than horizontal. - Second position in vertical layout with flickering effect attracted the most attention and sales. - 25% of products added to carts were influenced by the recommendation interface.	- Non-random participant selection limits generalizability. - Fixed sequence of layouts may introduce bias. - No consideration of eye fatigue or cognitive processes. - Results specific only to furniture shopping, which may not generalize to other types of products.
De Leon-Martinez et al. (2023) [34]	- The least restrictive areas of interest (AOI) threshold resulted in the best performance, improving the recall and ranking of selected movies compared to the baseline click-only models. - Movies that received longer fixation times were more likely to be included in the recommendation model, leading to better alignment between user interest and recommendations.	- The study was limited by the small size of the dataset and the specific setup, which may affect the generalizability of the findings. - The study did not implement a probabilistic click model, which could have further validated the impact of eye tracking data on improving recommendation accuracy. - The study focused on a single type of media (movies) and a specific interface design (circular list), which may limit the applicability of the findings to other types of recommender systems and user interfaces.

## Data Availability

Not applicable. This study does not report any data.
